# Brincidofovir Effectively Inhibits Proliferation of Pseudorabies Virus by Disrupting Viral Replication

**DOI:** 10.3390/v16030464

**Published:** 2024-03-18

**Authors:** Huihui Guo, Qingyun Liu, Dan Yang, Hao Zhang, Yan Kuang, Yafei Li, Huanchun Chen, Xiangru Wang

**Affiliations:** 1 National Key Laboratory of Agricultural Microbiology, College of Veterinary Medicine, Huazhong Agricultural University, Wuhan 430070, China; guohuihui@webmail.hzau.edu.cn (H.G.); liuqy@mail.hzau.edu.cn (Q.L.); danyang6211@webmail.hazu.edu.cn (D.Y.); 2022302110193@webmail.hzau.edu.cn (H.Z.); ky19981459286@163.com (Y.K.); lyfovlyf@163.com (Y.L.); chenhch@mail.edu.cn (H.C.); 2 Key Laboratory of Preventive Veterinary Medicine in Hubei Province, The Cooperative Innovation Center for Sustainable Pig Production, Huazhong Agricultural University, Wuhan 430070, China; 3 Frontiers Science Center for Animal Breeding and Sustainable Production, Huazhong Agricultural University, Wuhan 430070, China; 4 International Research Center for Animal Disease, Ministry of Science and Technology of China, Wuhan 430070, China

**Keywords:** pseudorabies virus, human infection, antiviral drugs, brincidofovir

## Abstract

Pseudorabies is an acute and febrile infectious disease caused by pseudorabies virus (PRV), a member of the family Herpesviridae. Currently, PRV is predominantly endemoepidemic and has caused significant economic losses among domestic pigs. Other animals have been proven to be susceptible to PRV, with a mortality rate of 100%. In addition, 30 human cases of PRV infection have been reported in China since 2017, and all patients have shown severe neurological symptoms and eventually died or developed various neurological sequelae. In these cases, broad-spectrum anti-herpesvirus drugs and integrated treatments were mostly applied. However, the inhibitory effect of the commonly used anti-herpesvirus drugs (e.g., acyclovir, etc.) against PRV were evaluated and found to be limited in this study. It is therefore urgent and important to develop drugs that are clinically effective against PRV infection. Here, we constructed a high-throughput method for screening antiviral drugs based on fluorescence-tagged PRV strains and multi-modal microplate readers that detect fluorescence intensity to account for virus proliferation. A total of 2104 small molecule drugs approved by the U.S. Food and Drug Administration (FDA) were studied and validated by applying this screening model, and 104 drugs providing more than 75% inhibition of fluorescence intensity were selected. Furthermore, 10 drugs that could significantly inhibit PRV proliferation in vitro were strictly identified based on their cytopathic effects, virus titer, and viral gene expression, etc. Based on the determined 50% cytotoxic concentration (CC_50_) and 50% inhibitory concentration (IC_50_), the selectivity index (SI) was calculated to be 26.3–3937.2 for these 10 drugs, indicating excellent drugability. The antiviral effects of the 10 drugs were then assessed in a mouse model. It was found that 10 mg/kg brincidofovir administered continuously for 5 days provided 100% protection in mice challenged with lethal doses of the human-origin PRV strain hSD-1/2019. Brincidofovir significantly attenuated symptoms and pathological changes in infected mice. Additionally, time-of-addition experiments confirmed that brincidofovir inhibited the proliferation of PRV mainly by interfering with the viral replication stage. Therefore, this study confirms that brincidofovir can significantly inhibit PRV both in vitro and in vivo and is expected to be an effective drug candidate for the clinical treatment of PRV infections.

## 1. Introduction

Pseudorabies virus (PRV) belongs to the family Herpesviridae, the subfamily Alphaherpesvirus, and the genus Varicella. It is enveloped and harbors a double-stranded DNA genome. PRV infection has been reported in a wide range of mammals, including pigs, cattle, sheep, cats, dogs, and other domestic animals, as well as wild animals. Among them, pigs are the exclusive natural reservoir of PRV [1]. After being infected, newborn piglets suffer from fatal encephalitis, resulting in 100% mortality, breeding pigs exhibit reproductive disorders, and fattening pigs experience stunted growth. PRV infection in other susceptible animals is characterized by severe pruritus and central nervous system (CNS) dysfunction, ultimately resulting in 100% mortality [2]. Apparently, PRV is neurotropic and lethal for a wide range of hosts.

The global pig industry has suffered significant economic losses due to the high prevalence of PRV. Despite vaccination and eradication measures, PRV remains endemic in wild boars and domestic pigs worldwide, posing a major threat to pig farming as one of the most important animal infectious diseases. China has experienced two outbreaks of porcine pseudorabies caused by classical and variant strains, respectively [3]. Several studies have demonstrated that the virulence of variant PRV strains is significantly enhanced compared to classical strains [4]. These variant PRV strains have been circulating in Chinese pig populations since 2011.

It has long been controversial as to whether humans can be infected with PRV. In 1914, two researchers who were studying PRV were suspected of being infected with PRV after coming to contact with contaminated materials and exhibited symptoms such as weakness, agitation, sore throat, and pruritus [5]. From then until 1992, there were 17 reported cases of suspected human infection with PRV [6].

From 2017 to the present, there have been a total of 30 clinical cases of PRV infection in humans reported in China, all of which were classified as pseudorabies encephalitis. All patients exhibited fever and neurological symptoms such as seizures and impaired consciousness. Over half of the patients experienced severe visual impairment, presenting as acute retinal detachment, vitreous clouding or blindness. Of the 30 reported cases, 28 individuals were involved in industries related to pig production and pork marketing, including veterinarians, butchers, and pork salesman. Next-generation sequencing (NGS) revealed variable reads and coverage of PRV sequences in the tissues of these patients, while some patients also exhibited detectable levels of PRV antibodies in their sera [2,7,8,9,10,11,12,13,14,15]. In addition, we previously isolated the PRV strain hSD-1/2019 from the cerebrospinal fluid of a patient, providing direct pathogenetic evidence to support PRV infection in humans [2]. After receiving aggressive treatment, unfortunately, the majority of these patients had a poor prognosis, with neurological sequelae or even death. Currently, vaccination against pseudorabies is the primary effective measure for preventing and controlling porcine pseudorabies. However, comprehensive treatment is typically employed for human pseudorabies encephalitis, as there are currently no specific antiviral drugs available for PRV infection. Due to the disabling and lethal effects of pseudorabies on both animals and humans, it is imperative to identify effective antiviral drugs for controlling PRV infection.

Some natural products have been reported to exhibit anti-PRV activity, such as isobavachalcone [16], (-)-epigallocatechin-3-gallate [17], resveratrol [18], kaempferol [19], and quercetin [20], etc. These drugs exhibited potential antiviral effects in vivo or in vitro; however, their inhibitory effects on PRV proliferation were mostly observed at high effective concentrations. The effective concentration of (-)-epigallocatechin-3-gallate and kaempferol in inhibiting PRV was as high as 50 μM in vitro and 240 mg/kg in vivo, respectively. Hydroquinone and adefovir dipivoxil, approved by the U.S. Food and Drug Administration (FDA), have demonstrated certain antiviral efficacy against classical PRV strains; however, their effectiveness against prevalent variant PRV strains in China and human-origin PRV strains remains uncertain [21,22]. So far, all of the studies on the development of anti-PRV drugs have been in the phase of laboratory screening and validation.

In cases of human pseudorabies encephalitis caused by PRV infection, patients were administered commonly used anti-herpesvirus drugs (such as acyclovir, ganciclovir, and penciclovir) along with other medical interventions. Despite treatment, several patients succumbed to the disease, while all surviving patients experienced varying degrees of neurological sequelae. Most importantly, the inhibitory effect of acyclovir on the human-origin PRV strain hSD-1/2019 was evaluated at different drug concentrations. The results indicated that in vitro, acyclovir did not significantly inhibit the virus and failed to provide adequate protection for challenged mice. In this study, a high-throughput screening method for anti-PRV drugs was established and utilized to screen 2104 FDA-approved drugs. Brincidofovir was identified as having notable antiviral effects against hSD-1/2019 in vitro and in vivo, providing 100% protection for lethally challenged mice. This effective anti-PRV drug is of great significance for the clinical treatment of PRV infection.

## 2. Materials and Methods

### 2.1. Cells and Viruses

Porcine kidney cells (PK-15) were purchased from the China Center for Type Culture Collection (CCTCC) and preserved in our laboratory. PK-15 cells were grown in Dulbecco’s modified Eagle’s high-glucose medium (DMEM) supplemented with 10% fetal bovine serum (FBS) (GIBCO, Grand Island, NY, USA) at 37 °C with 5% CO_2_. The classical PRV strain Ea was isolated from the infected pig and the variant PRV strain hSD-1/2019 was isolated from cerebrospinal fluid of the infected patient. These PRV strains were isolated and preserved in our laboratory [2].

### 2.2. Construction of Fluorescently Labeled Viruses

The fluorescence-labeled viruses were constructed and rescued by inserting mCherry in front of the terminator of the PRV UL35 gene through homologous recombination. Briefly, the genomic DNA of hSD-1/2019 and Ea strains was extracted using TIANamp Genomic DNA Kit (TIANGEN, Beijing, China). Polymerase chain reactions (PCRs) were then conducted to amplify the homologous sequences and the red fluorescent protein expressing gene mCherry with primers listed in Table 1. The homologous recombinant transfer plasmids were constructed by sequentially inserting these sequences into pcDNA3.1 (+) vector (Biofeng, Shanghai, China). The correct homologous recombinant plasmids were named as pcDNA3.1 (+)-mCherry-hSD and pcDNA3.1 (+)-mCherry-Ea, respectively.

The combined linear fragments of the homologous arms and mCherry were amplified from the recombinant plasmid and purified. The purified fragments were transfected into PK-15 cells in a 6-well plate using Lipofectamine^®^ 2000 Reagent (Life, Carlsbad, CA, USA) according to manufacturer’s instructions. At 4 h post transfection, the cells were infected with hSD-1/2019 or Ea at a multiplicity of infection (MOI) of 0.001 and then incubated at 37 °C with 5% CO_2_ for 1–2 days. During the incubation, red fluorescent cytopathic effects (CPEs) were observed. When the cells showed 80% CPEs, the culture was collected and a “freeze–thaw cycle” was conducted three times. After that, virus plaque purification was performed 5 times to obtain the recombinant mutants hSD-mCherry and Ea-mCherry as previously described [2].

### 2.3. Cytotoxicity Test

The cytotoxicity of the drugs on PK-15 cells was assessed with the Cell Counting Kit-8 assay (CCK-8) according to the instructions provided by the manufacturers of the CCK-8 kit (Beyotime, Shanghai, China). Briefly, the drug was diluted to 320, 160, 80, 40, 20, and 10 μM, respectively, and added to PK-15 cells at 80% confluence in a 96-well plate. Six replicate wells were set for each dilution as well as the cell control without drug treatment. After 36 h of incubation at 37 °C with 5% CO_2_, 10 μL of CCK-8 reagent was added per well and the plate was then incubated for another 1 h. Absorbance values at 450 nm were measured. The viability of cells treated with drugs was calculated according to the following formula: Average absorbance value (cells treated with the drug)/Average absorbance value (cell control). Nonlinear regression (curve fitting) analysis was then conducted to obtain the 50% cytotoxicity concentration 50% (CC_50_), which was defined as the drug concentration that reduced cell viability by 50% when compared to untreated controls.

### 2.4. Half Maximal Inhibitory Concentration (IC_50_) Determination

The IC_50_ of the drugs against PRV was assessed in PK-15 cells using the CCK-8 assay. In a 96-well plate, PK-15 cells monolayers at 80% confluence were treated with drugs at different concentrations and infected with hSD-1/2019 at 0.01 MOI. After 36 h of incubation at 37 °C with 5% CO_2_, 10 μL of CCK-8 reagent was added per well. Absorbance values at 450 nm were measured after another 1 h of incubation. The inhibition ratio of each drug against PRV was calculated according to the following formula: (Average absorbance value (cells infected with PRV) − Average absorbance value (cells treated with drugs and infected with PRV))/Average absorbance value (cell infected with PRV). The IC_50_ was illustrated by means of nonlinear regression analysis using GraphPad Prism, indicating the concentration of the drug that inhibited virus replication by 50%.

### 2.5. The 50% Tissue Culture Infectious Dose (TCID_50_) Assay

The virus titer was determined by means of TCID_50_ assay as previously described, with some modifications [23]. Then, 100 μL of a PK-15 cell suspension containing 2 × 10^4^ cells was added to each well. Negative controls containing only PK-15 cells were set up in two columns. The virus solution was 10-fold diluted serially from approximately 10^−1^ to 10^−8^ in FBS-free DMEM medium, and 100 μL of each dilution was added into a 96-well plate with 8 replicates. The 96-well plates were incubated at 37 °C with 5% CO_2_ for 7 days, and CPEs were checked daily and recorded. After the observation, TCID_50_ was calculated according to the Reed–Muench method.

### 2.6. Quantitative PCR (qPCR) Detecting gE Gene

PRV *gE* gene copies were detected by means of TaqMan qPCR as previously described [2]. The standard recombinant plasmid was prepared and the standard curve was constructed. Viral DNA was extracted from the samples using the TIANamp Genomic DNA Kit (TIANGEN, Beijing, China) and the *gE* gene of the extracted DNA was amplified by TaqMan qPCR. The *gE* copies were calculated according to the detected cycle threshold value of the sample and the standard curve.

### 2.7. One-Step Growth Curve

Confluent monolayer PK-15 cells were infected with PRV at a dose of 0.1 MOI followed by incubation at 37 °C with 5% CO_2_ for 2 h. After incubation, the medium was discarded and cells were washed twice with PBS before adding fresh DMEM supplemented with 3% FBS (the maintenance medium). Both cells and supernatant were collected at 0, 4, 8, 12, 16, 20, 24, 28, 32, 36, 40 and 48 h post incubation in the maintenance medium. After three freeze–thaw cycles, the supernatant of lysates was collected via centrifugation and titrated as TCID_50_ in PK-15 cells.

### 2.8. In Vivo Assessment of Antiviral Effects of the Drugs

The antiviral effects of the drugs were assessed in mice, which was conducted in the Experimental Animal Center of Huazhong Agricultural University (animal welfare assurance number HZAUMO-2022-0143). Mice were randomly grouped, with 5 mice in each group. Except for 5 mice injected with DMEM as a blank control, other mice were challenged with hSD-1/2019 at a dose of 500 TCID_50_ via hind footpad injection. Treatment was started at the same time as the challenge, and all drugs were injected intraperitoneally using a dose of 10 mg/kg/d for 5 days. Five challenged mice were set as the hSD-1/2019 control with intraperitoneal injection of DMEM. The clinical symptoms and mortality of the mice were observed daily and recorded for 14 days. The clinical symptoms of mice in each group were scored according to the scoring criteria in Table 2.

Upon the onset of mortality in the hSD-1/2019 control group, two mice were humanely euthanized and dissected. Additionally, two mice from both the Brincidofovir administration group and the blank control group were also euthanized and dissected. The brain, lung, and spleen tissues were collected, fixed in 4% paraformaldehyde (Biosharp, Beijing, China), and sent for the preparation of sections with hematoxylin-eosin (HE) staining at HYcell Biotechnology Co., Ltd. (Wuhan, China).

### 2.9. Time-of-Addition Assay

The time-of-addition assay was performed to determine the stage of viral replication cycle targeted by the drug by adjusting the order in which the drug and viruses were added into cells. During all the experiments, monolayer PK-15 cells in 96-well plates were infected with PRV-mCherry at an MOI of 0.01 and treated with the drug at 10 μM. (I) Virus inactivation: The mixture of viruses and drug was incubated at 37 °C for 1 h and then added to the cells for continued incubation. After 36 h, the cultures were collected. (II) Pre-treatment effect: A concentration of 10 μM of the drug was added to the cells for 1 h of incubation at 37 °C and then replaced by the viral suspension. The cells were covered with fresh medium 1 h post infection and incubated for 36 h before harvesting. (III) Virus internalization: The cells were infected with PRV-mCherry and incubated at 4 °C for 1 h. Then, the supernatant was replaced with the drug, followed by 1 h of incubation at 37 °C. The drug was removed and fresh medium was added. The cultures were collected after 36 h. (IV) Virus replication: The cells were infected with PRV-mCherry and incubated at 37 °C for 1 h. Then, the supernatant was replaced with the drug and the cells were incubated at 37 °C for 36 h before collection. These collected cultures were applied for fluorescence intensity detection and virus titer determination.

### 2.10. Statistical Analysis

Data are presented as the mean ± standard deviation (SD) from 3 independent experiments. GraphPad Prism version 6.0 software (GraphPad, La Jolla, CA, USA) was used for statistical analysis. The significant difference between groups was analyzed using Student’s *t*-test or two-way ANOVA. A level of 0.01 < *p* < 0.05 (*) was considered significant, *p* < 0.01 (**) or *p* < 0.001 (***) was considered statistically highly significant and extremely significant, respectively. *p* > 0.05 (ns) was considered not significant.

## 3. Results

### 3.1. Fluorescence Intensity of the Recombinant PRV-mCherry Could Indicate Virus Proliferation Titers

**Figure 1:**
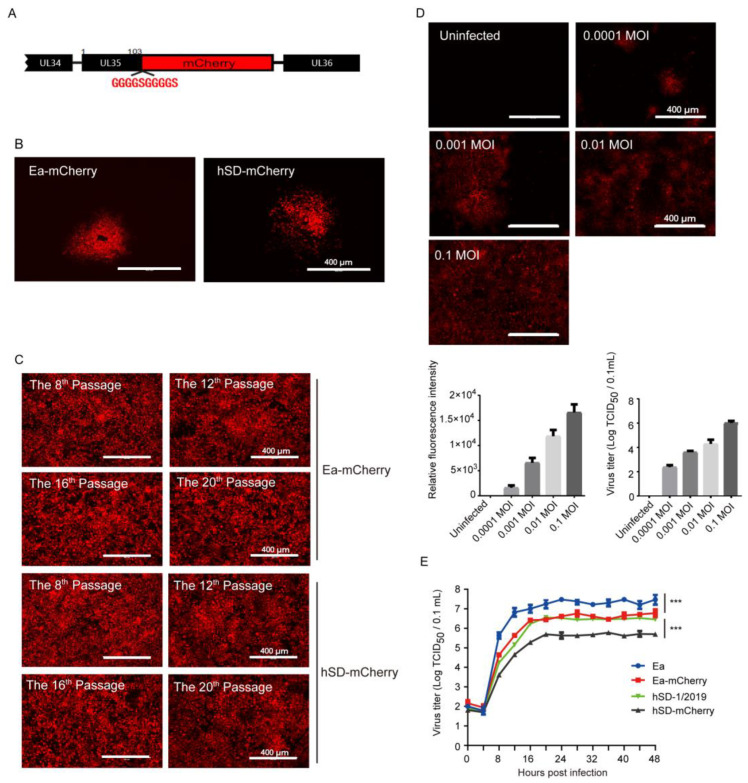
The PRV strains labeled with mCherry could express the protein stably and exhibited a proliferation trend similar to that of the wild-type strains. (**A**) The coding sequences of mCherry were fused upstream of the terminator region of PRV UL35 gene to generate PRV-mCherry strains; (**B**) plaque purification of recombinant Ea-mCherry and hSD-mCherry; (**C**) Ea-mCherry and hSD-mCherry were passaged in PK-15 cells and their fluorescence intensity of the 8th, 12th, 16th, and 20th passages were observed and compared; (**D**) the PK-15 cells were infected with PRV-mCherry at different MOI. Fluorescence intensity and virus titers were measured at 24 hpi, revealing a similar increasing trend; (**E**) one-step growth curves of PRV-mCherry strains and wild-type strains in PK-15 cells with an MOI of 0.01. Virus titers between groups were analyzed by Student’s *t*-test. A level of *p* < 0.001 (***) was considered statistically extremely significant.

The recombinant PRV strains hSD-mCherry and Ea-mCherry, which were fluorescently labeled, were generated by inserting the mCherry gene upstream of the terminator sequence within the UL35 gene of PRV (Figure 1A). After plaque purification, virions with red fluorescence were obtained (Figure 1B). The recombinant hSD-mCherry and Ea-mCherry were passaged in PK-15 cells, and the fluorescence intensity of the culture in different passages was found to be comparable, indicating a consistent expression of mCherry during virus replication (Figure 1C). With an increasing infection dose, the fluorescence intensity and virus titer of cell culture exhibited a parallel increase (Figure 1D), indicating that PRV-mCherry’s fluorescence intensity can serve as a direct indicator of the virus proliferation titer. Additionally, the one-step growth curves of WT PRV strains and mCherry-labeled strains were comparable, despite the lower virus titers observed in the recombinant strains compared to their parental WT counterparts (Figure 1E). These findings suggest that PRV-mCherry strains can be effectively utilized for subsequent drug screening.

### 3.2. The Anti-Herpesvirus Drugs Commonly Used in Clinical Practice Exhibited Limited Efficacy against the PRV Variant Strain hSD-1/2019

**Figure 2:**
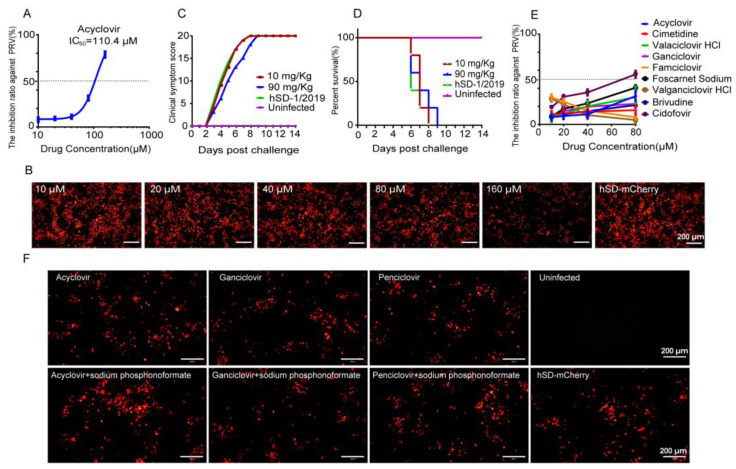
The commonly used clinical anti-herpesvirus drugs exhibited limited efficacy against the PRV variant strain hSD-1/2019. (**A**) IC_50_ of acyclovir against PRV. The dotted line represents 50% inhibition; (**B**) PK-15 cells were infected with hSD-mCherry at 0.01 MOI and treated with different concentrations of acyclovir. Fluorescence intensity was observed at 48 hpi; (**C**) clinical symptom scores of the mice according to the scoring criteria in Table 2. The data are presented as the total daily score for each group of mice; (**D**) survival curves of the mice. Daily mortality rates for each group were recorded over a period of 14 days, and survival curves were plotted using GraphPad Prism; (**E**) inhibition ratio of nine anti-herpesvirus drugs at different concentrations against PRV. PK-15 cells were infected with hSD-mCherry at 0.01 MOI and treated with drugs at various concentrations. The fluorescence intensity was measured by a multimode microplate reader at 48 hpi, and drug inhibition ratios were calculated using the following formula: (Fluorescence intensity [cells infected with PRV]—Fluorescence intensity [cells treated with drug and infected with PRV])/(Fluorescence intensity [cells infected with PRV]) × 100%; (**F**) PK-15 cells were infected with hSD-mCherry at 0.01 MOI and treated with drugs individually or in combination groups consisting of treatment with either 10 μM acyclovir, ganciclovir, or penciclovir alone, or combined treatment involving both antiviral drugs and sodium phosphonoformate at a concentration of 10 μM each, respectively. Fluorescence intensity was observed at 48 hpi, showing similar fluorescence intensities across all groups.

Of the 30 previously reported PRV-infected patients, 15 received antiviral medication containing acyclovir, 5 received antiviral medication containing ganciclovir, and 1 received antiviral medication containing penciclovir. However, the efficacy of these drugs against PRV remains to be studied given their poor final outcomes [24]. Subsequently, the efficacy of several clinical anti-herpesvirus medications against the human-origin PRV variant strain hSD-1/2019 were evaluated in this study. The IC_50_ of acyclovir against hSD-1/2019 was determined to be 110.4 μM (Figure 2A). At a concentration of 160 μM, acyclovir significantly attenuated the fluorescence intensity of PK-15 cells infected with hSD-mCherry compared to the lower concentrations (Figure 2B). However, noticeably, the IC_50_ values of acyclovir against herpes simplex virus type-1 and Varicella Zoster virus were both less than 10 μM [25,26], indicating much higher susceptibility to acyclovir than the PRV variant strain hSD-1/2019. The anti-PRV effect of acyclovir was further investigated in mice challenged with a lethal dose of hSD-1/2019. Mice that received no medication showed severe neurological symptoms and 100% mortality, while those treated with acyclovir at doses of 10 mg/Kg and 90 mg/Kg had the same clinical symptom scores and mortality rates as the untreated group, indicating that acyclovir did not protect mice from lethal PRV infection (Figure 2C,D).

Furthermore, the anti-PRV effects of nine anti-herpesvirus drugs were additionally assessed in vitro. The results revealed that cidofovir at a concentration of 80 μM exhibited an inhibition rate of 56% against PRV, while the remaining eight drugs demonstrated less than 50% inhibition, even at their highest tested concentrations (80 μM) (Figure 2E). The clinical application of combining antiviral drugs with sodium phosphonoformate has been commonly employed to enhance the antiviral effect. Therefore, we added sodium phosphonoformate to test the antiviral effects of acyclovir, ganciclovir, and penciclovir. The fluorescence intensity of the combined medication groups did not show any significant differences compared to that of the individual medication groups or the non-medicated group (Figure 2F). The aforementioned data indicate that the clinically utilized anti-herpesvirus drugs, including acyclovir, exhibit ineffectiveness against the human-origin PRV strain hSD-1/2019 both in vitro and in vivo. Therefore, there is an imperative and urgent need to conduct a comprehensive screening for efficacious anti-PRV drugs to combat PRV infection.

### 3.3. Eighteen Drugs Effectively Inhibiting PRV-mCherry Proliferation Were Screened out from 2104 FDA-Approved Drugs through the High-Throughput Screening Method

**Figure 3:**
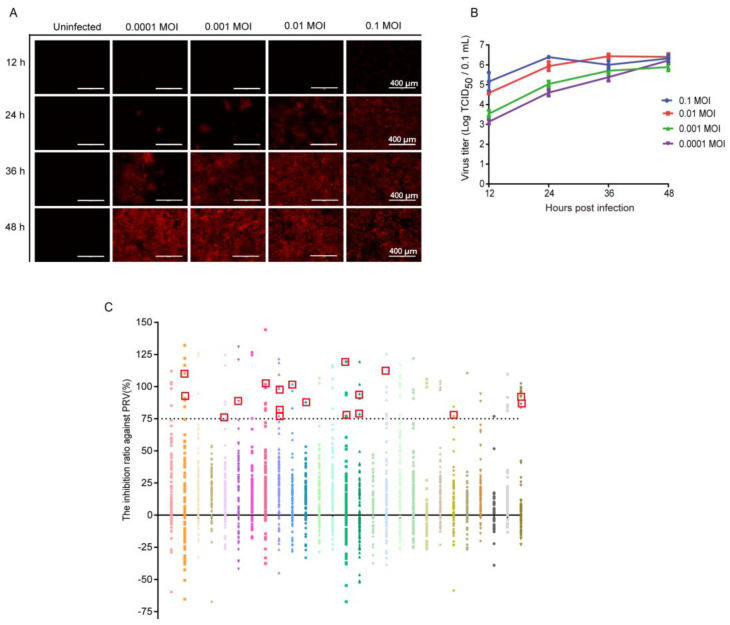
A high-throughput method for screening anti-PRV drugs was developed, resulting in the identification of 18 out of 2104 FDA-approved drugs based on this approach. (**A**,**B**) PK-15 cells were infected with Ea-mCherry at different dose. The fluorescence intensity and virus titer of each group was observed and determined at 12, 24, 36, and 48 hpi, respectively. The virus titer is expressed as the mean ± standard deviation of 3 independent analyses. In cells infected with Ea-mCherry at 0.01 MOI for 36 h, the fluorescence intensity was the strongest, while the virus titer reached its highest level; (**C**) PK-15 cells were infected with Ea-mCherry at 0.01 MOI and were treated with the drug at a concentration of 10 μM. After incubation for 36 h, the inhibition ratio of the drugs against PRV was calculated according to the following formula: (Fluorescence intensity _(cells infected with PRV)_ − Fluorescence intensity _(cells treated with drug and infected with PRV)_)/Fluorescence intensity _(cells infected with PRV)_ × 100%. A total of 2104 drugs were tested here. Each color represents a parallel screening of 96-well plate. The Y axis shows the inhibition rate of drugs on PRV calculated as described above. Each symbol represents a kind of drug. The symbols above the dotted line at “75” represent the drugs showing an inhibition ratio against PRV greater than or equal to 75%, 18 drugs (red squares) were confirmed to effectively reduce the CPEs induced by virus infection without exhibiting obvious cytotoxic effects on the cells. These drugs are considered as effective anti-PRV drugs in vitro. Data are expressed as the mean ± standard deviation of 3 independent analyses.

A high-throughput method was developed based on the fluorescently labeled PRV strains described above. The optimization results for inoculation dose and infection time demonstrated that cells infected with Ea-mCherry at 0.01 MOI for 36 h exhibited peak fluorescence intensity and virus titers, which remained stable (Figure 3A,B). Accordingly, the established high-throughput method for drug screening involved infecting PK-15 cell monolayers in 96-well plates with Ea-mCherry at 0.01 MOI, followed by treatment with a 10 μM drug. Cells infected with PRV served as the infection control group. After incubation for 36 h, the fluorescence intensity of cells was measured using a multimode microplate reader instrument with excitation light at 587 nm and emission light at 610 nm. The inhibition ratio of the drug against Ea-mCherry was calculated as the percentage difference in fluorescence intensity between infected cells and drug-treated cells.

Through this high-throughput screening method, a total of 2104 drugs approved by the FDA for the market and clinical disease treatment were tested (Figure 3C). The dotted line at “75” in Figure 3C is the threshold for judgement of effective inhibition on PRV proliferation. A drug was considered to inhibit PRV proliferation effectively if its inhibition ratio against PRV was greater than or equal to 75%. Each symbol represents a kind of drug. As illustrated in Figure 3C, 104 drugs exhibited the inhibition ratio against PRV higher than 75%. Subsequently, the cell morphology of each group was individually observed, and 18 out of the 104 drugs were confirmed to effectively reduce the CPEs induced by virus infection without exhibiting obvious cytotoxic effects on the cells. The names of the 18 selected drugs are shown in Table 3. They are tanespimycin (17-AAG), ganetespib (STA-9090), triapine, topotecan HCl, floxuridine, amonafide, TAS-102, adefovir dipivoxil, trifluridine, tenofovir alafenamide hemifumarate, tenofovir alafenamide fumarate, baricitinib phosphate, pixantrone Maleate, cerdulatinib (PRT062070), camptothecin, cytarabine hydrochloride, methotrexate, and brincidofovir.

### 3.4. In Vitro Evaluation of the Anti-PRV Drug Candidates

**Figure 4:**
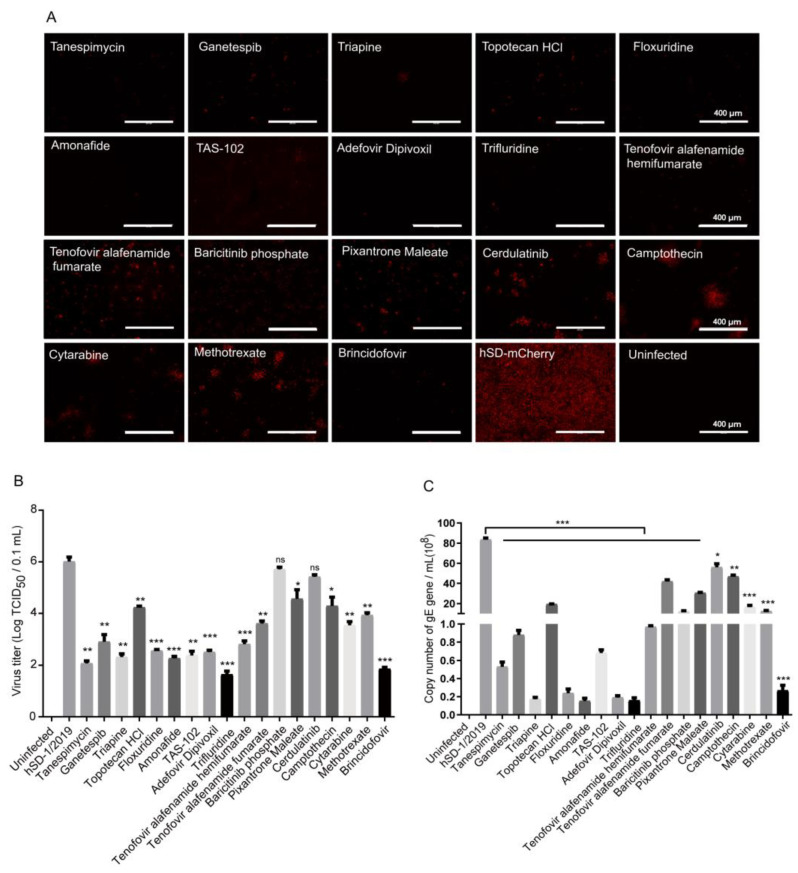
The inhibitory activity of 18 candidate drugs against PRV was evaluated in vitro. (**A**) PK-15 cells were infected with hSD-mCherry at an MOI of 0.01 and treated with the 18 drugs at a concentration of 10 μM. After 36 h of infection, the fluorescence intensity of the cells was observed; (**B**,**C**) viral gE gene copies and virus titers were quantified using TaqMan qPCR and TCID_50_ determination, respectively. All data were presented as the mean ± standard deviation from three independent analyses. Statistical analysis using Student’s *t*-test was performed to determine significant differences between the hSD-mCherry-infected group and the medication group. A level of 0.01 < *p* < 0.05 (*) was considered significant, and *p* < 0.01 (**) or *p* < 0.001 (***) was considered statistically highly significant and extremely significant, respectively. *p* > 0.05 (ns) was considered not significant.

The inhibitory effects of the 18 screened drug candidates on hSD-mCherry proliferation were evaluated in vitro. All 18 drugs demonstrated significant reductions in fluorescence intensity, viral gE gene copies, and virus titers in cells infected with hSD-mCherry (Figure 4A–C). The top 10 drugs exhibiting the highest inhibition of viral gE gene expression and virus titers were selected for further testing. The CC_50_ values of these 10 drugs ranged from 22.32 to 1124.00 μM, while their IC_50_ values against PRV ranged from 0.075 to 4.076 μM (Table 4, Figures S1 and S2). Consequently, all of these selected drugs exhibited a calculated select index (SI) greater than or equal to 25, indicating their potential as effective anti-PRV agents (Table 4).

### 3.5. In Vivo Evaluation of the 10 Drug Candidates

**Figure 5:**
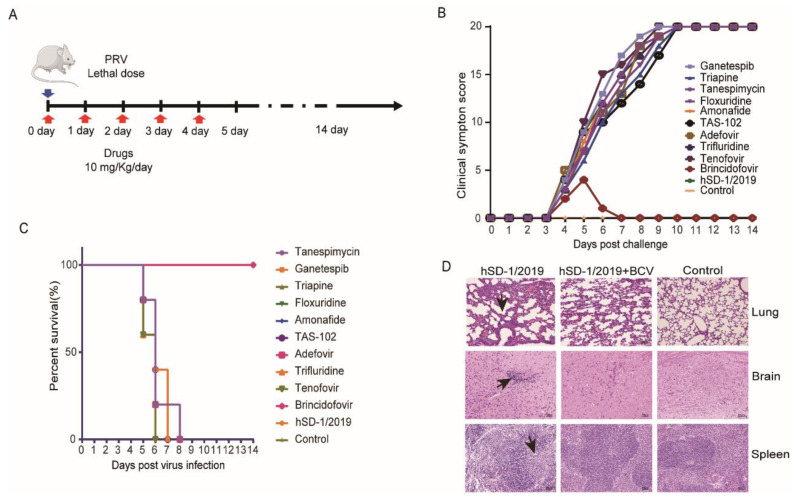
Therapeutic effect of the 10 drugs in mice infected with a lethal dose of hSD-1/2019 (**A**) Schematic diagram of the mouse model. The red arrows indicate drug medication and the blue arrow indicates the virus challenge. Mice were challenged with PRV hSD-1/2019 at 500 TCID_50_ via hind foot pad injection. The medication groups were intraperitoneally treated with the drugs at a daily dose of 10 mg/Kg for five days. The observation period lasted for 14 days; (**B**) clinical symptoms in each group were scored daily and the total score of mice in a group is displayed, where higher scores indicate more severe clinical symptoms. A score of 20 represents death of all 5 mice in a group; (**C**) survival curve analysis was performed using Kaplan–Meier survival plots to evaluate the survival rates among different groups. The color lines represent different drug treatment groups; (**D**) histopathological examination was conducted on lung, brain and spleen tissues collected from three groups when mice died in the hSD-1/2019 group.

The 10 drug candidates showing significant in vitro anti-PRV effects were further assessed in mice to validate their therapeutic efficacy against PRV infection in vivo. As shown in Figure 5A, mice were exposed to hSD-1/2019 at a lethal dose of 500 TCID_50_ and intraperitoneally administered the candidate drug at a dose of 10 mg/kg for 5 consecutive days. Starting from the fourth day post infection, severe symptoms including abdominal scratching, persistent gnawing on the hind injected limbs leading to bone mutilation and tissue necrosis, and mortality (Figure 5B) were observed in both the hSD-1/2019 group and most drug groups. In contrast, mice treated with brincidofovir only exhibited mild clinical signs, such as bedding rubbing and abdominal scratching, on day 4 after infection, which completely disappeared by day 7 (Figure 5B). Consistently, survival curve analysis demonstrated that brincidofovir could provide complete protection against lethal hSD-1/2019 infection, while all mice in the other medication groups succumbed to the infection (Figure 5C). These findings suggest that brincidofovir exhibits comprehensive protective effects in hSD-1/2019-infected mice.

The brain, lung, and spleen tissues were collected from mice in the brincidofovir medication group, the hSD-1/2019 group, and the blank control group for section preparation and histopathological analysis. In the hSD-1/2019 group, the lung tissues of mice exhibited pulmonary congestion, thickened alveolar septa, enlarged alveolar cavities that fused to form large pulmonary alveoli, and significant intra-alveolar inflammatory cell infiltration. Massive monocyte infiltration was observed in the brain tissues. The spleen showed the disappearance of acini lienalis and blurred boundaries between red and white marrow (left column images of Figure 5D). Compared to the hSD-1/2019 group, mice treated with brincidofovir displayed reduced pulmonary congestion and alveolar septum thickening, more intact alveolar profiles, and a smaller amount of inflammatory cell infiltration in the alveolar lumen. No pathological changes were observed in the brain or spleen tissues (middle column images of Figure 5D). None of the tissues from mice in the blank control group showed any pathological changes (right column images of Figure 5D). Therefore, brincidofovir demonstrated a significant therapeutic effect on mice with lethal PRV infection.

### 3.6. Brincidofovir Inhibits Virus Proliferation Mainly by Interfering with the Viral Replication Phase

**Figure 6:**
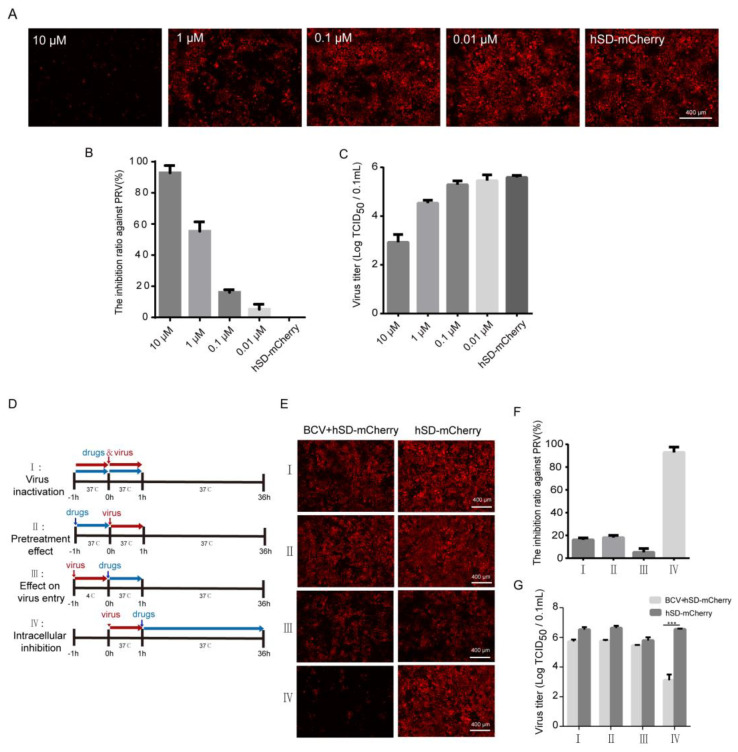
The stage of BCV inhibiting PRV virus infection. (**A**–**C**) PRV was inoculated into an 80% confluence of PK-15 cells at the dose of 0.01 MOI, and different concentrations of BCV were added. After incubation for 24 h, the fluorescence intensity was measured to calculate the inhibition rate of PRV based on the fluorescence intensity. Simultaneously, the virus titers were determined at different drug concentrations; (**D**) schematic illustration of the time-of-addition experiment; (**E**–**G**) resuscitated PK-15 cells were cultured in a cell incubator to form a monolayer for subsequent use. PRV virus infection and drug treatment followed a time-of-addition test diagram approach. The inhibition rate of drugs at different stages of PRV replication was assessed using fluorescence intensity measurements, and further confirmed by detecting the viral titer. A level of *p* < 0.001 (***) was considered statistically extremely significant.

We subsequently evaluated and verified the antiviral efficacy of brincidofovir against PRV virus proliferation. The findings demonstrate a significant dose-dependent reduction in PRV virus titer upon treatment with brincidofovir (Figure 6A–C). To further elucidate the specific stage of the PRV life cycle targeted by brincidofovir, time-of-addition assays were conducted (Figure 6D). Following the experimental protocol outlined in the Section 2, a concentration of 10 μM of brincidofovir was used along with a virus dose of 0.1 MOI. The results revealed that group IV treated with brincidofovir exhibited significantly lower fluorescence intensity compared to the control group (Figure 6E), indicating an enhanced inhibitory effect on viral replication (Figure 6F) and a substantial decrease in virus titer (Figure 6G). These observations suggest that brincidofovir primarily acts during the replication stage of PRV. However, no notable changes were observed in terms of the virus fluorescence intensity or titer in groups I, II, and III, implying that direct virucidal activity or interference with PRV adsorption or internalization is not among the mechanisms employed by brincidofovir.

## 4. Discussion

PRV infection is currently epidemic in various areas worldwide with dense pig populations, including Asia, South America, and Europe. Although pseudorabies has been successfully eliminated from domestic pigs in some countries, there is still a risk of virus transmission to domestic pig populations from wild animals, particularly wild boars [27,28]. Pseudorabies is one of the major infectious diseases in China, with a seropositivity rate of 35% in the last five years in 24 provinces. PRV infection has caused substantial economic losses to the Chinese pig farming industry. Vaccination has been the primary method of controlling and eliminating PRV infection in pigs [29]. As a typical alpha herpesvirus, PRV can build latent infection and be reactivated under certain stimulations. The reactivated virus can spill out and cause infection in pig herds. Moreover, latent PRV cannot be detected and therefore cannot be eliminated in time [1]. Vaccination cannot prevent PRV from establishing latent infection nor block the virus from spilling out after infection [30]. Effective anti-PRV drugs can potentially suppress latent infection and virus reactivation, preventing the virus from spilling out, which is of great importance for pseudorabies elimination in pigs and the treatment of human pseudorabies encephalitis.

Since 2017, 30 cases of PRV infection in humans have been reported in China. The human-origin PRV strain hSD-1/2019 was previously isolated from the cerebrospinal fluid of a patient in our laboratory. It was highly genomically and biologically similar to the PRV variant strains currently prevalent in pigs in China, suggesting the potential transmission risk of the PRV variant strains from pigs to humans [2]. The current worldwide spread of PRV in pigs has increased the contact degree between the virus and humans, promoting its potential to become a zoonotic disease and highlighting its threat to public health. In the 30 cases of human pseudorabies encephalitis, all patients developed neurological symptoms soon after the onset of febrile symptoms. After the treatment, five patients ultimately died and most survivors suffered from sequelae, including a coma (7/30), visual impairment (13/30), and delayed responses (4/26) [24]. The antiviral treatment of the patients did not prevent the rapid progression of neurological damage. PRV usually causes fatal infections in non-natural hosts, and the infected animals die within 24 h of developing neurological signs [31]. Therefore, early and effective antiviral treatment is particularly important for preventing neurological dysfunction and death caused by PRV infection.

The commonly used clinical anti-herpesvirus drugs include acyclovir, ganciclovir, famciclovir, valaciclovir, cidofovir, penciclovir, valganciclovir, trifluridine, vidarabine, cytarabine, and edoxudine [32]. These drugs are mostly nucleosides or nucleotide analogs, which could inhibit or interfere with the synthesis of viral DNA [33]. Antiviral drugs including acyclovir, ganciclovir, and penciclovir were administered to the 30 patients with a PRV infection after the initial diagnosis of viral encephalitis. Considering the poor progression of the patients, the antiviral effect of these drugs on PRV remained to be evaluated. Here, it was found that these clinically used anti-herpesvirus drugs showed few antiviral effects against the human-origin PRV strain hSD-1/2019 in vitro and in vivo (Figure 2), suggesting the urgency of developing more effective anti-PRV drugs. 

It was reported that resveratrol showed effective anti-PRV effects with an IC_50_ of 17.17 μM in vitro [34], and 30 mg/kg resveratrol could completely protect piglets intranasally infected with PRV at a dose of 2 × 10^6^ TCID_50_ [18]. However, the antiviral mechanism of these natural products is unclear and requires more research before clinical application. Hydroquinone and adefovir dipivoxil, which are listed in the FDA-approved drug library, were found to have a significant antiviral effect in vitro and in vivo against classical PRV strains, while the effects against the variant PRV strains and human-origin strains are unclear [21,22]. Although the valproic acid derivative valpromide (VPD) could inhibit PRV proliferation in PK-15 and Neuro-2a cells in the concentration range of 0.5 to 1.5 mM, no data support its medicative effect in vivo [35]. Ivermectin, an antiparasitic drug, also exhibited 100% inhibition of PRV replication at 2.0 μM in vitro and reduced mortality by 50% in PRV-infected mice with attenuated brain damage [23].

According to the results in Figure 6, it can be suggested that brincidofovir primarily acts during the replication stage of PRV. However, it remains unclear as to which specific events in the PRV replication cycle are regulated by brincidofovir. Brincidofovir is a lipid conjugate of the acyclic nucleotide phosphonate cidofovir (CDV) [36]. The lipid side chain of brincidofovir hydrolyzes in cells to release CDV, which is then phosphorylated into CDV-diphosphate (CDV-DP). This compound inhibits the DNA polymerase enzyme that aids in viral DNA synthesis and suppresses viral DNA replication by disrupting the function of viral DNA polymerase and destabilizing viral DNA [37,38]. The addition of a lipid moiety increases the bioavailability and half-life while reducing nephrotoxic side effects associated with brincidofovir [39]. Therefore, brincidofovir is more effective in vivo than many other antiviral drugs. Based on these previous studies, the inhibition of brincidofovir on PRV DNA replication will be focused on in future investigations. At present, this drug is undergoing clinical trials for the treatment of patients severely infected with DNA viruses, including cytomegalovirus [40], adenovirus [38], herpes simplex virus [41], polyomavirus [42], smallpox virus [43], and monkeypox virus [44]. Moreover, it has been approved by the FDA for the treatment of smallpox virus [36], highlighting its high potential as a clinical medication in humans. In this study, brincidofovir was identified as a significant antiviral drug against both classical and variant PRV strains in vitro and in vivo. The IC_50_ of brincidofovir against PRV was remarkably low, at 0.5439 μM (Table 4), while the administration of 10 mg/kg brincidofovir provided complete protection to mice from fatal infections caused by the human-origin variant PRV strain hSD-1/2019 (Figure 5). Since 2017, there has been an increasing number of reported cases involving human pseudorabies encephalitis. This emerging viral encephalitis poses serious risks such as severe neurological damage, death, and long-term complications, even with adequate clinical treatments, thus necessitating further attention towards elucidating its pathogenic mechanisms and developing effective antiviral drugs through future research. 

## 5. Conclusions

In this study, we assessed the inhibitory efficacy of commonly utilized anti-herpesvirus medications (e.g., acyclovir, etc.) against PRV strains and observed their limited effectiveness. Herein, we developed a high-throughput screening method for identifying PRV antiviral drugs based on fluorescently labeled PRV strains and a multimode microplate reader that quantified fluorescence intensity to depict virus proliferation. A total of 2104 small-molecule drugs approved by the FDA were screened and validated using this model. Remarkably, the continuous administration of brincidofovir at a dosage of 10 mg/kg for 5 days provided complete protection in mice challenged with a lethal dose of a human-origin PRV strain. Experimental evidence confirmed that brincidofovir primarily impedes viral replication, thereby significantly inhibiting PRV both in vitro and in vivo. Consequently, these findings establish brincidofovir as a promising drug candidate for the clinical treatment of PRV infection.

## Figures and Tables

**Table 1:** Primers used for PCR in this study.

Target Sequences	Primer	Sequences (5′-3′)
Upstream homologous arm	PRV-HindIII-F	CCCAAGCTTAGGCCGCGTACCCTCCG
	PRV-KpnI-R	CGGGGTACCGGGCGAGGGGCGAGGG
mCherry	mCherry-KpnI-F	CGGGGTACCGGTGGAGGCGGTTCAGGCGGAG GTGGCTCTATGGTGAGCAAGGGCGAGGA
	mCherry-BamHI-R	CGCGGATCCCTTGTACAGCTCGTCCATGC
Downstream homologous arm	PRV-BamHI-F	CGCGGATCCTAGCCCCGCGCGATCAATAAAG
	PRV-EcoRI-R	CCGGAATTCCCGCGCGTGGTGGAGTCG

**Table 2:** Scoring criteria of clinical symptoms in mice.

Score	Clinical Symptoms
0	No symptoms
1	Excitement, restlessness, occasional itching and scratching
2	Ataxia, severe itching, persistent gnawing on hind limbs
3	Gnawing on the hind limbs resulting in bone disruption and tissue necrosis
4	Dead or dying

**Table 3:** The 18 drugs effectively inhibiting PRV and screened out by the high-throughput method.

Number	Drug	Target
1	Tanespimycin (17-AAG)	Cytoskeletal Signaling
2	Ganetespib (STA-9090)	Cytoskeletal Signaling
3	Triapine	DNA/RNA Synthesis
4	Topotecan HCl	DNA Damage
5	Floxuridine	DNA Damage
6	Amonafide	DNA Damage
7	TAS-102	DNA/RNA Synthesis
8	Adefovir Dipivoxil	Microbiology
9	Trifluridine	DNA Damage
10	Tenofovir alafenamide hemifumarate	Reverse Transcriptase
11	Tenofovir alafenamide fumarate	Reverse Transcriptase
12	Baricitinib phosphate	JAK/STAT
13	Pixantrone Maleate	DNA Damage
14	Cerdulatinib (PRT062070)	JAK/STAT
15	Camptothecin	Topoisomerase
16	Cytarabine hydrochloride	DNA Damage
17	Methotrexate	Metabolism
18	Brincidofovir	DNA/RNA Synthesis

**Table 4:** CC_50_, IC_50_ and SI of 10 drug candidates.

Number	Drug	CC_50_ (μM)	IC_50_ (μM)	SI (CC_50_/IC_50_)
1	Tanespimycin (17-AAG)	48.89	0.46	106.08
2	Ganetespib (STA-9090)	294.51	0.07	3937.17
3	Triapine	78.66	2.99	26.29
4	Floxuridine	375.43	0.23	1653.74
5	Amonafide	241.71	1.24	194.76
6	TAS-102	1124.00	0.95	1186.66
7	Adefovir Dipivoxil	227.61	4.08	55.84
8	Trifluridine	352.40	1.43	246.09
9	Tenofovir hemifumarate	225.95	2.79	81.09
10	Brincidofovir	22.32	0.54	41.04

## Data Availability

Data are contained within the article and Supplementary Materials.

## Supplementary Materials

The following supporting information can be downloaded at: https://www.mdpi.com/article/10.3390/v16030464/s1, Figure S1: The 50% cytotoxic concentration (CC_50_) measurement of 10 drugs. Figure S2: Half maximal inhibitory concentration (IC_50_) of the 10 candidate drugs.
