# Supplementary material for: Brincidofovir Effectively Inhibits Proliferation of Pseudorabies Virus by Disrupting Viral Replication
Source: Viruses. 2024 Mar 18;16(3):464. doi: 10.3390/v16030464 (PMC10975951; doi:10.3390/v16030464)
Supplement: Supplementary file 1 [file viruses-16-00464-s001.zip › viruses-2883254-supplementary.pdf]

Supplementary Material

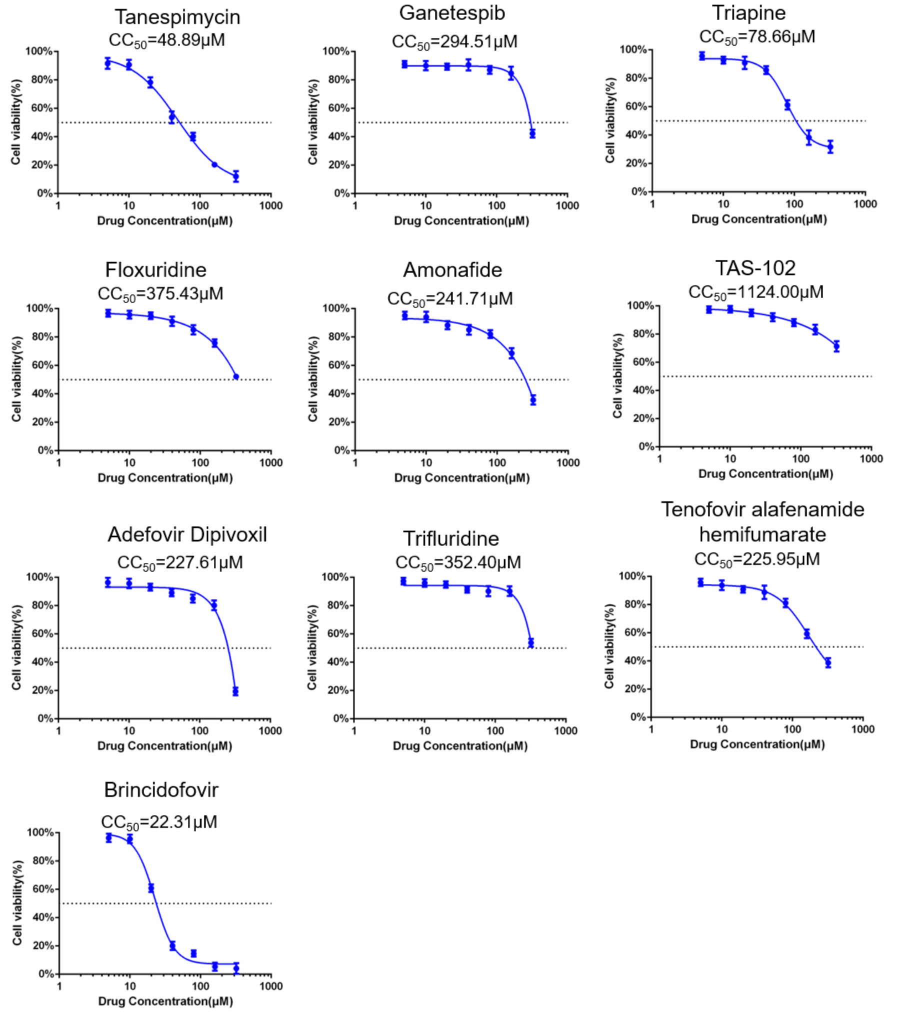

Figure S1. The 50% cytotoxic concentration (CC_{50}) measurement of 10 drugs. 10 candidate drugs were diluted to 320, 160, 80, 40, 20, and 10 μM, respectively, and added to PK-15 cells at 80% confluence in a 96-well plate. 6 replicate wells were set for each dilution as well as the cell control without drug treatment. After 36 h incubation at 37°C and 5% CO_2, 10 μL CCK-8 reagent per well was added and the plate was then incubated for another 1 h. Absorbance values at 450 nm were measured. Viability of cells treated with drugs was calculated according to the formula: Average absorbance value (cells treated with the drug) / Average absorbance value (cell control). Nonlinear regression (curve fitting) analysis was then conducted to obtain the concentration cytotoxicity 50% (CC_{50}).

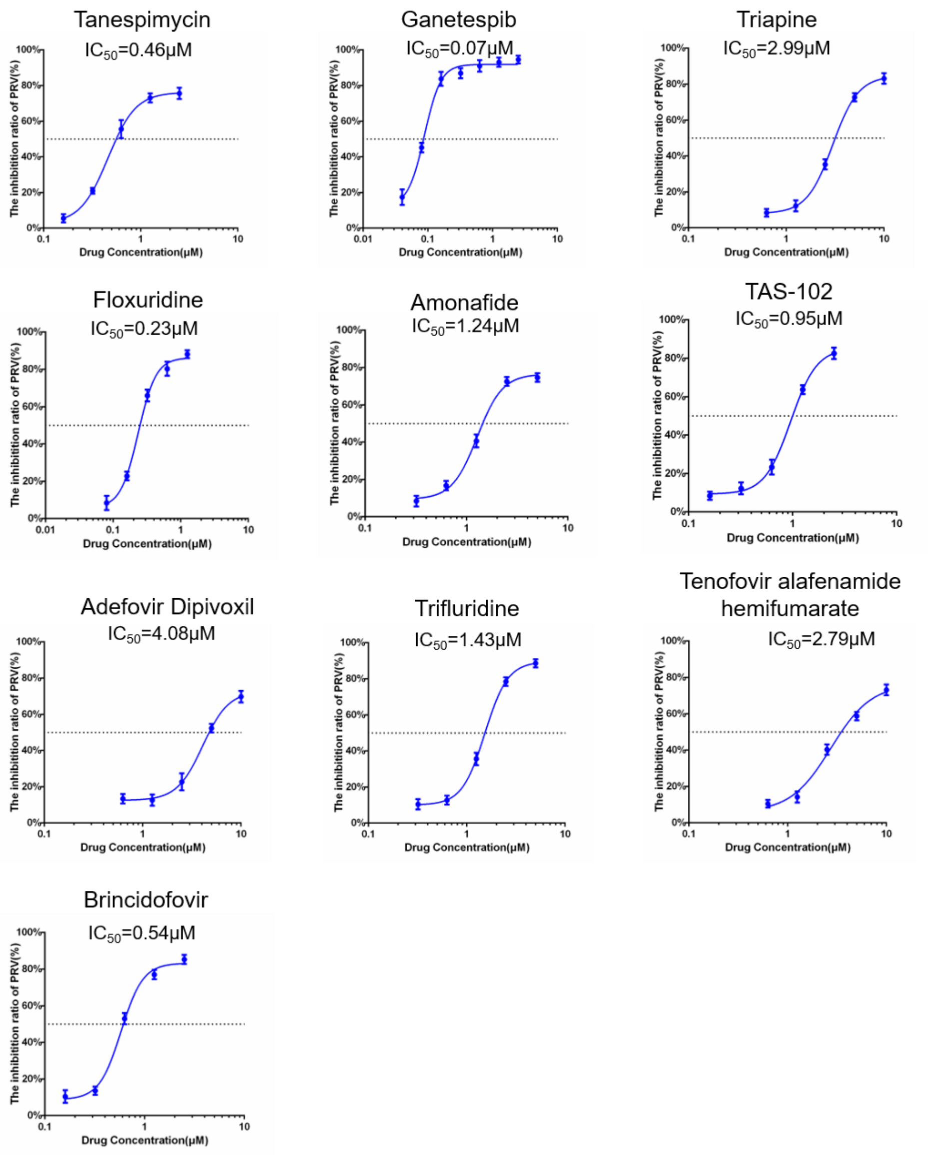

Figure S2. Half maximal inhibitory concentration (IC_{50}) of the 10 candidate drugs. In a 96-well plate, PK-15 cells monolayers at 80% confluence were treated with drugs at different concentrations and infected with hSD-1/2019 at 0.01 MOI. After 36 h incubation at 37°C and 5% CO_2, 10 μL CCK-8 reagent per well was added. Absorbance values at 450 nm were measured after another 1 h incubation. The inhibition ratio of drug against PRV was calculated according to the formula: (Average absorbance value (cells infected with PRV) – Average absorbance value (cells treated with drugs and infected with PRV)) / Average absorbance value (cell infected with PRV). IC_{50} was illustrated by nonlinear regression analysis using GraphPad Prism.
